# Knockout of *Foxp2* disrupts vocal development in mice

**DOI:** 10.1038/srep23305

**Published:** 2016-03-16

**Authors:** Gregg A. Castellucci, Matthew J. McGinley, David A. McCormick

**Affiliations:** 1 Yale School of Medicine, Department of Neuroscience, New Haven, 06519, USA; 2 Yale University, Department of Linguistics, New Haven, 06520, USA; 3 Haskins Laboratories, New Haven, 06511, USA

## Abstract

The *FOXP2* gene is important for the development of proper speech motor control in humans. However, the role of the gene in general vocal behavior in other mammals, including mice, is unclear. Here, we track the vocal development of *Foxp2* heterozygous knockout (Foxp2+/−) mice and their wildtype (WT) littermates from juvenile to adult ages, and observe severe abnormalities in the courtship song of Foxp2+/− mice. In comparison to their WT littermates, Foxp2+/− mice vocalized less, produced shorter syllable sequences, and possessed an abnormal syllable inventory. In addition, Foxp2+/− song also exhibited irregular rhythmic structure, and its development did not follow the consistent trajectories observed in WT vocalizations. These results demonstrate that the *Foxp2* gene is critical for normal vocal behavior in juvenile and adult mice, and that *Foxp2* mutant mice may provide a tractable model system for the study of the gene’s role in general vocal motor control.

The *FOXP2* gene encodes a transcription factor critical to the development of normal speech motor control in humans^1^,^2^,^3^,^4^,^5^,^6^. Mutations in a single copy of the gene result in Developmental Verbal Dyspraxia (also referred to as Childhood Apraxia of Speech), a severe speech−language disorder characterized by an inability to coordinate the rapid and precise articulatory movements executed during speech production^7^,^8^. *FOXP2* sequence and expression is highly conserved throughout vertebrates^9^,^10^, suggesting a similar, albeit not language specific, function for the gene across species. Instead, *FOXP2* may be important for general orofacial motor coordination and vocal behavior, as is suggested by previous reports demonstrating disruptions in the vocalizations of songbirds following *FoxP2* knockdown^11^,^12^, and changes in *FoxP2* expression in the songbird brain coupled with periods of song learning^13^ and song production^14^.

To further investigate the role of the gene in mammals, several lines of *Foxp2* mutant mice have been generated, including *Foxp2* knockout (KO) mice^15^, mice carrying deleterious *Foxp2* mutations analogous to those observed in humans^16^,^17^, and mice possessing a humanized *FOXP2* gene^18^. While studies of these mutant mice revealed interesting alterations in anatomy and synaptic plasticity in both the striatum and cerebellum, examinations of their vocalizations did not provide a clear understanding for the role of *Foxp2* in mouse vocal behavior. For example, while mice homozygous for *Foxp2* KO or deleterious *Foxp2* mutations display stark abnormalities in their innate distress and isolation calls as pups^15^,^16^,^17^,^18^,^19^, these animals die from severe motor and respiratory defects before adulthood^15^,^20^, confounding the observed vocal phenotypes. Meanwhile, the heterozygotes of these mouse lines produce pup vocalizations with either small, or no apparent, differences from wildtype mice^15^,^16^,^17^,^18^,^19^. In addition, mice possessing a humanized *FOXP2* gene produce vocalizations exhibiting minor alterations as pups only^18^, and their adult vocalizations are no different from wildtype mice^21^.

This study aims to further investigate the role of the *Foxp2* gene in mouse vocal behavior by examining the effects of *Foxp2* KO on the production of adult male courtship vocalizations^22^ (song), rather than pup calls. Specifically, we assess the song production of *Foxp2* heterozygous knockout mice^15^ and their wildtype littermates from weaning to adulthood, and observe severe abnormalities in both the production and development of *Foxp2* heterozygous KO mouse song.

## Results and Discussion

In order to examine possible alterations in the courtship song of *Foxp2* heterozygous knockout (Foxp2+/−) mice, we recorded the vocalizations of five litters of Foxp2+/− mice (n = 11 mice) and their wildtype (WT) littermates (n = 14). From weaning (~P23) to adulthood (~P75), each male mouse was socialized with a random WT female mouse 2–3 times a week for at least 3 minutes. During the socialization sessions, the ultrasonic vocalizations produced were recorded and analyzed (see Methods and Supplementary Tables S1–S3 for methodological and statistical details, respectively).

### Song Production

**Figure 1:**
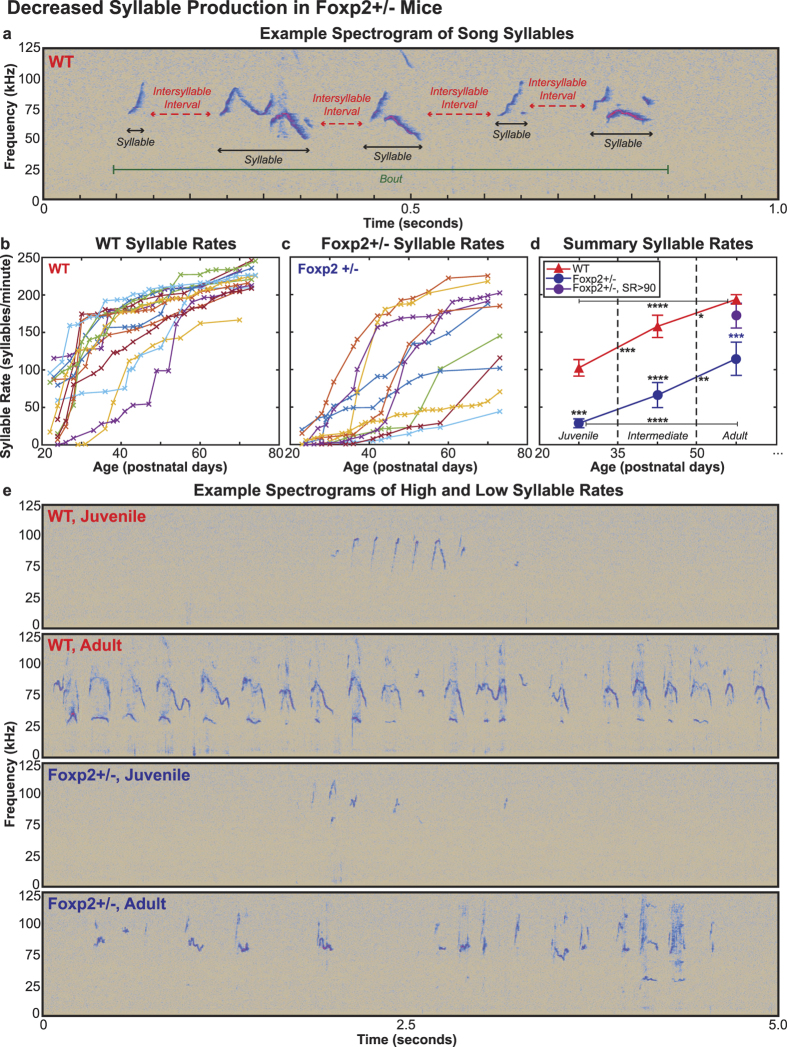
Decreased Syllable Production in Foxp2+/− Mice. (**a**) Example spectrogram of song syllables from an adult WT animal with syllables, intersyllable intervals, and bout duration labelled. (**b**,**c**) Syllable rate (syllables per minute) in each recording session for (**b**) WT and (**c**) Foxp2+/− mice. Each line represents a different animal. (**d**) Mean age group syllable rate for WT (red) and Foxp2+/− (blue) mice. Data are presented as means +/− 1 standard error of the mean (SEM). All significant differences are indicated (*p < 0.05; **p < 0.005; ***p < 0.0005; ****p < 0.0001; two-way repeated measures ANOVA with Sidak’s Correction for multiple comparisons, except for comparisons between adult age groups, where ordinary one-way ANOVA with Tukey’s Correction for multiple comparisons is used). Between-genotype significant differences are indicated above the Foxp2+/− data points; blue asterisks indicate significant differences between WT adult and Foxp2+/− adult groups. All other asterisks indicate significant differences between age groups of the same genotype. Additional statistical details are presented in Supplementary Table S1. (**e**) Example spectrograms of song from juvenile and adult WT (top and second from top, respectively) and Foxp2+/− mice (second from bottom and bottom, respectively), demonstrating differences in syllable rate.

Mouse song, like other rodent ultrasonic vocalizations, consists of bouts of calls separated into syllables by periods of silence^23^ (Fig. 1a). We defined syllables as calls separated by at least 40 ms of silence, as we found that there was a clear trough in the distribution of these silent durations at this value (Supplementary Figure S1d). This finding is consistent with previous results from an analysis of breathing during vocalization^24^, which showed that mice produce one syllable per breath, and delineating syllables by silent periods of less than ~40 ms resulted in incorrectly splitting individual calls produced on a single breath into separate syllables.

We first examined how vigorously each mouse vocalized by calculating syllable rate (SR; the number of syllables produced per minute) in each recording, and we found that WT mice produced higher syllable rates with age (Fig. 1b,d,e, Supplementary Table S1) and that all WT mice produced more than 90 syllables per minute by P50. Furthermore, in order for male mice to consistently produce this high syllable rate, we found it is critical that they be socialized starting at a young age, as mice socialized starting at older ages were more variable in their vocalization production (Supplementary Figure S2).

While Foxp2+/− mice also produced more song with age (Fig. 1c–e, Supplementary Table S1), their syllables rates were more variable and several mice produced less than 90 syllables per minute even at the oldest age recorded. To allow for an analysis of vocal development, we binned the data from each mouse into three age groups: 1) adult (P50–P75), as WT mice stably produced more than 90 syllables per minute by this age, 2) intermediate (P35–P49), motivated by the fact that mice begin to develop a sex instinct and produce mature spermatozoa around P35^25^, and 3) juvenile (P20–P34). Calculating an average syllable rate for each age group revealed that both WT and Foxp2+/− mice produced significantly more song with age, and that WT mice produced more song than Foxp2+/− mice at every age (Fig. 1d, Supplementary Table S1).

As it is possible that Foxp2+/− mice produced less song on average than their WT littermates because of motivational or other social behavioral alterations, we formed an additional group of Foxp2+/− mice consisting of adults whose syllable rates were greater than 90 syllables per minute (Foxp2+/− (SR > 90), n = 6). As expected, the syllable rate of the Foxp2+/− (SR > 90) group of mice was not significantly different from the adult WT group (Fig. 1d, Supplementary Table S1). Therefore, this group allowed us to control for potential motivational differences between the genotypes as both the WT adult and Foxp2+/− (SR > 90) adult mice were equally motivated to sing.

### Syllable Duration

**Figure 2:**
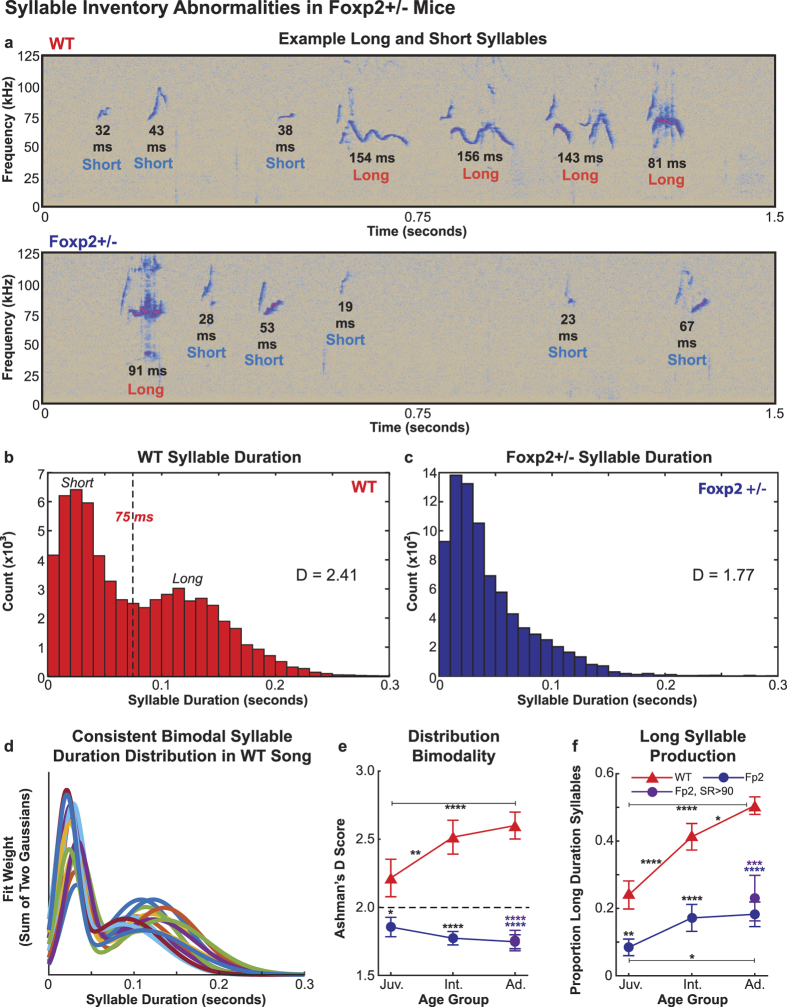
Syllable Inventory Abnormalities in Foxp2+/− Mice. (**a**) Example spectrograms of short and long syllables in WT adult song (top), and Foxp2+/− adult song (bottom). (**b,c**) Histograms of syllable duration from all age groups for (**b**) WT, and (**c**) Foxp2+/− mice. For (**b**) and (**c**), the number of syllable duration measures contributed by individual mice has been randomly down-sampled so that each animal provides an equal proportion of data points to the overall genotype sample. Ashman’s D scores for the genotype-wide distributions appear on the right side of each histogram. (**d**) Sum of the fit of two Gaussians to each WT mouse’s syllable duration distribution across all ages; each line represents a different animal. (**e**) Average Ashman’s D scores across animals for both genotypes at each age. (**f**) Average proportion of long syllables across animals for both genotypes at each age. In (**e**) and (**f**), data are presented as means +/− 1 SEM. All significant differences are indicated (*p < 0.05; **p < 0.005; ***p < 0.0005; ****p < 0.0001; two-way repeated measures ANOVA with Sidak’s Correction for multiple comparisons, except for comparisons between adult age groups, where ordinary one-way ANOVA with Tukey’s Correction for multiple comparisons is used). Between-genotype significant differences are indicated above the Foxp2+/− data points; blue and purple asterisks indicate significant differences between WT adult and Foxp2+/− adult groups and WT adult and Foxp2+/− (SR > 90) adult groups, respectively. All other asterisks indicate significant differences between age groups of the same genotype. Additional statistical details are presented in Supplementary Table S1.

Humans with mutations in the *FOXP2* gene have been noted to display strong speech timing and prosodic abnormalities^4^. To determine whether the vocalizations of Foxp2+/− mice also possess temporal irregularities, we next examined syllable duration as a gross ‘prosodic’ metric of mouse song. We found that WT mice produced syllables whose duration was bimodally distributed, with one peak at approximately 25 ms and second at 130 ms (Fig. 2a,b). This bimodal distribution of syllable durations was a consistent feature of WT song, and was observed in the song of every WT mouse we examined (Fig. 2d). Based on the position of the trough in the genotype-wide bimodal distribution, we selected a 75 ms cut-off to operationally define “short” (<75 ms) and “long” (>75 ms) duration syllables (Fig. 2a,b).

In contrast to their WT littermates, Foxp2+/− mice lacked long syllables as a distinct class, though they still produced some syllables longer than 75 ms (Fig. 2a,c). To assess the strength of the bimodality in the WT distributions and the lack of bimodality in the Foxp2+/− distributions, we fit each animal’s syllable duration distribution with two Gaussians and computed genotype-wide average Ashman’s D scores across animals. This metric quantifies the degree of separation between two Gaussian distributions, with a score greater than 2 signifying clear separation; for example, the Ashman’s D score for the WT distribution in Fig. 2b is 2.41, but only 1.77 for the Foxp2+/− distribution in Fig. 2c. We found that the average Ashman’s D score for WT mice was significantly greater than that of the Foxp2+/− mice at every age, and that the average score was greater than 2 for only the WT mice (Fig. 2e, Supplementary Table S1). Furthermore, we also found that the Ashman’s D score significantly increased with age only in the WT mice (Fig. 2e, Supplementary Table S1), which resulted from a significant increase in the duration of long syllables with age in that genotype (Supplementary Figure S3a, Supplementary Table S1).

We next quantified the production of long syllables (>75 ms in duration) over time, and found that WT animals produced significantly more long syllables with age (Fig. 2f, Supplementary Table S1). This result was consistent whether the 75 ms cutoff was used, or the average Gaussian fit weight of the long syllable distribution was analyzed over time (Supplementary Figure S3b, Supplementary Table S1). The Foxp2+/− mice also produced more long syllables with age, but the increase was only significant when comparing the juvenile production rates to the adult production rates (Fig. 2f, Supplementary Table S1). Furthermore, WT mice were also found to produce significantly more long syllables than Foxp2+/− mice at every developmental stage (Fig. 2f, Supplementary Table S1).

### Acoustic Properties

Having determined that WT mice produce two classes of syllables that are *temporally* distinct, we next examined whether short and long syllables differ in their spectral structure by measuring four acoustic features of adult song: 1) frequency modulation within a syllable (standard deviation of the dominant frequency of a syllable), 2) amplitude modulation within a syllable (standard deviation of the amplitude of a syllable), 3) the number of pitch jumps in a syllable (dominant frequency changes within a syllable of >10 kHz from one time point to the next), and 4) the overall dominant frequency of the syllable. We found that long syllables were significantly more frequency modulated and amplitude modulated than short syllables, that long syllables contained significantly more pitch jumps than short syllables, and that long syllables were produced with a lower dominant frequency than short syllables (Table 1, Supplementary Table S3).

Since short and long syllables have different acoustic signatures, we compared the acoustic structure of WT short and long syllables directly to Foxp2+/− short and long syllables rather than grouping the two heterogeneous syllable classes together. Despite the lack of syllable duration bimodality, we applied the 75 ms cutoff to delineate long and short syllables in the Foxp2+/− data as we wanted to determine whether the longer duration syllables produced by the Foxp2+/− mice were acoustically irregular. To ensure that any differences we observed were not due to alterations in motivation, we compared WT adults to the Foxp2+/− (SR > 90) group only, and found that there were no significant differences between the two genotypes in the acoustic structure of short or long syllables (Table 2, Supplementary Table S3). This finding suggests that, in agreement with Hammerschmidt *et al.* (2015), *Foxp2* expression is not required for the production of *acoustically* normal syllables. The gene’s expression instead appears to be important for producing syllables with the proper *temporal* features. Therefore, while the Foxp2+/− mice are capable of producing short and long syllables with the WT-like acoustic features, they appear to be unable to produce long syllables with the proper temporal features that clearly distinguish them from short syllables.

### Sequence Production

**Figure 3:**
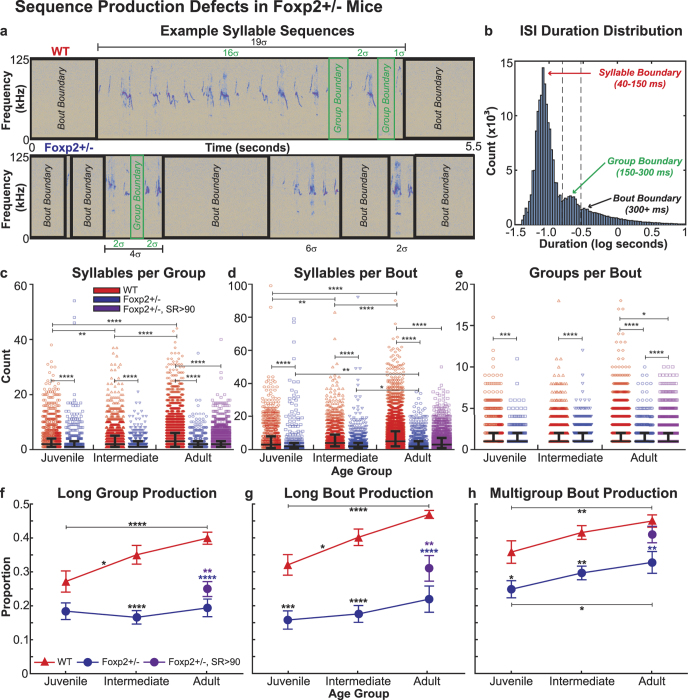
Sequence Production Defects in Foxp2+/− Mice. (**a**) Example spectrograms of syllable sequences from adult WT (top) and Foxp2+/− (bottom) animals. Each group boundary, bout boundary, and the number of syllables in every group and bout is labelled. (**b**) Histogram of intersyllable interval (ISI) duration for all animals. The ISI distributions corresponding to syllable boundaries (40–150 ms), group boundaries (150–300 ms), and bout boundaries (300+ ms) are labeled. (**c–e**) Distributions of the number of (**c**) syllables per group, (**d**) syllables per bout, and (**e**) groups per bout for both genotypes at each age group, with the medians and first and third quartile values indicated. In (**c**–**e**) the number of sequencing measures contributed by individual mice has been randomly down-sampled so that each animal provides an equal proportion of data points to the respective total sample for each age group. (**f–h**) The average proportion of (**f**) long groups (>3 syllables), (**g**) long bouts (>5 syllables), and (**h**) multigroup bouts (>1 group) across animals for both genotypes at each age group. In (f) through (h), data are presented as means +/− 1 SEM. For (**c**) through (**h**), significance is reported as follows: *p < 0.05; **p < 0.005; ***p < 0.0005; ****p < 0.0001; using two-way repeated measures ANOVA with Sidak’s Correction for multiple comparisons, except for comparisons between adult age groups, where ordinary one-way ANOVA with Tukey’s Correction for multiple comparisons is used, and in (**c–e**) where Kruskal-Wallis tests with Dunn’s Correction for multiple comparisons are used. For (**f–h**), between-genotype significant differences are indicated above the Foxp2+/− data points; blue and purple asterisks indicate significant differences between WT adult and Foxp2+/− adult groups and WT adult and Foxp2+/− (SR > 90) adult groups, respectively. All other asterisks indicate significant differences between age groups of the same genotype. Additional statistical details are presented in Supplementary Tables S1 and S2.

We next examined how syllables were concatenated into sequences, as humans with *FOXP2* mutations display deficits in the sequencing of both oral and manual movements^7^,^26^. In order to determine natural sequence types, we first examined the distribution of the silent durations between syllables (intersyllable intervals; ISIs), as distinct sequence types may be separated by ISIs of different lengths. We examined the distribution of ISIs from all animals as we were unable to detect consistent differences between age groups and genotypes, in line with previous research reporting that ISI duration is stable across males of the same genotype that had undergone different experimental manipulations^27^. Our analysis revealed, in agreement with previous findings^28^, a non-uniform distribution (Fig. 3b). Based on the ISI distribution, we operationally defined three classes of ISI, which we term: 1) “syllable boundaries” (<150 ms), which separate individual syllables, 2) “group boundaries” (150–300 ms), which separate groups of syllables, and 3) “bout boundaries” (>300 ms), which separate bouts of groups (Fig. 3a,b). As mentioned previously, Sirotin *et al.* (2014) elegantly demonstrated that mice produce a single syllable with each breath, and that the breathing rate during vocalization is approximately 6 Hz. Interestingly, the result of not vocalizing on one breathing cycle would therefore be predicted to correspond to group boundaries, however further breathing analysis is required to confirm this hypothesis.

Both WT and Foxp2+/− mice were found to produce more syllables per group and per bout with age, though WT animals produced significantly more syllables in both sequence classes at all age groups (Fig. 3c,d, Supplementary Table S2). Differences between genotypes are especially clear when examining the production of long groups and bouts (groups and bouts containing more than the median number of syllables; 3 and 5, respectively), as WT mice produced approximately twice the proportion of both long sequence types in comparison to Foxp2+/− mice at every age. Furthermore, WT mice produced significantly more long groups and bouts with age, while Foxp2+/− animals showed no increase (Fig. 3f,g, Supplementary Table S1). The median number of groups per bout did not change significantly with age for either genotype (Fig. 3e, Supplementary Table S2), though the proportion of multigroup bouts (bouts consisting of 2 or more groups) produced did significantly increase with age (Fig. 3h, Supplementary Table S1). WT animals were found to produce significantly more groups per bout and multigroup bouts than Foxp2+/− mice (Fig. 3e,h, Supplementary Table S1, S2), although the deficit in multigroup production was no longer observed when adult WT mice were compared to adult Foxp2+/− (SR > 90) mice (Fig. 3h, Supplementary Table S1). This finding suggests that any deficits observed in Foxp2+/− mice in the structuring of groups into bouts is transient during development and minor in comparison to the production of long sequences of syllables.

### Song Rhythmicity

We investigated the rhythmic properties of WT and Foxp2+/− song by analyzing how short syllables, long syllables, group boundaries, and bout boundaries are temporally organized using conditional probabilities. Specifically, we calculated the probability of observing a short or long syllable 1) at the beginning of a group, 2) at the beginning of a bout, 3) at the end of a group, 4) at the end of a bout, 5) following a short syllable within a group, and 6) following a long syllable within a group (see Methods for more details). In order to make the most direct comparison between genotypes while also controlling for motivation, average conditional probabilities were compared between WT adult song and Foxp2+/− (SR > 90) adult song. As the Foxp2+/− mice still produced some syllables sufficiently long in duration to be considered long syllables by WT standards, we again separated long from short syllables in the Foxp2+/− data with the 75 ms cutoff. By using this cutoff, we were able to assess whether or not the Foxp2+/− animals produced their >75 ms long syllables in environments different than those where WT mice produced their long syllables.

**Figure 4:**
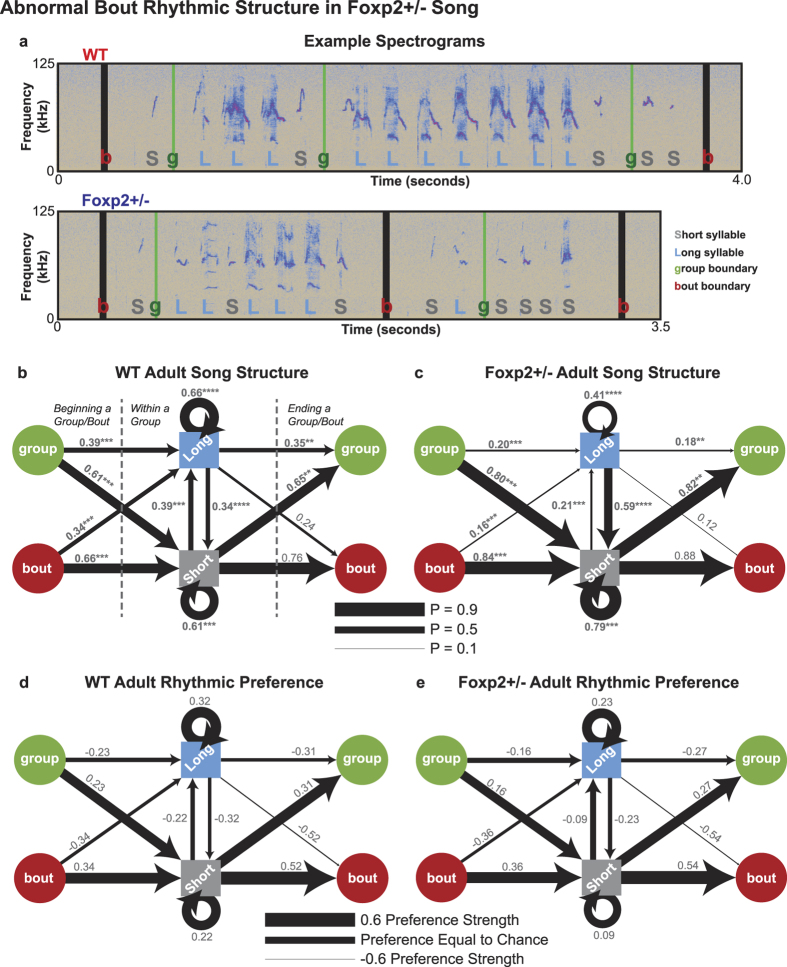
Abnormal Bout Rhythmic Structure in Foxp2+/− Song. **(a)** Example spectrograms of typical rhythmic structure in adult WT song (top) and adult Foxp2+/− song (bottom). Short syllables, long syllables, group boundaries, and bout boundaries are labelled as S, L, g, and b, respectively. **(b,c)** Average conditional transition probabilities of starting groups and bouts on either long or short syllables (left side of panel), ending groups and bouts on either short or long syllables (right side of panel), and producing short or long syllables given the production of a short or long syllable (center of panel) in (**b**) adult WT and (**c**) adult Foxp2+/− (SR > 90) song. Arrow weights indicate the magnitude of a transition’s probability. Significant differences in between genotypes are bolded. **(d,e)** Average Preference Strengths for the transitions in (**b**,**c**) in (**d**) adult WT and (**e**) adult Foxp2+/− (SR > 90) mice. Arrow weights indicate the magnitude of a transition’s Preference Strength. Significance is reported as follows: *p < 0.05; **p < 0.005; ***p < 0.0005; ****p < 0.0001; ordinary one-way ANOVA with Tukey’s Correction for multiple comparisons. Additional statistical details are presented in Supplementary Table S1.

We found that WT animals produced a simple stereotyped rhythmic pattern: bouts and groups were more likely to begin and end with short syllables, and both syllable classes were more likely to be produced in uninterrupted series as transitions between classes were less likely (Fig. 4a,b). The Foxp2+/− mice were found to produce the same general rhythmic pattern, however with a number of significant differences from WT (Fig. 4a,c). In general, the Foxp2+/− rhythmic pattern appeared more stereotyped than that of WT mice: groups and bouts were significantly more likely to start with a short syllable, groups were significantly more likely to end on a short syllable, and short syllables were significantly more likely to be produced in series. In addition, the Foxp2+/− mice were significantly more likely to transition from a long syllable to a short syllable, and therefore less likely to produce uninterrupted series of long syllables (Fig. 4c, Supplementary Table S1).

While the conditional probability analysis demonstrates the Foxp2+/− mice indeed produce rhythmically distorted song, it is unclear whether these observed abnormalities arise from the misapplication of an abstract rhythmic “rule” or instead is a byproduct of an abnormal syllable inventory. For instance, we have shown the Foxp2+/− mice produce a significantly lower proportion of long duration syllables than their WT littermates (Fig. 2e), and therefore produce proportionally more short syllables. Observing a higher conditional probability of producing uninterrupted series of short syllables may, for example, therefore be the result of an elevated probability of producing a short syllable by chance. To parse these two possibilities, we normalized the conditional probabilities in Fig. 4b,c by calculating average Preference Scores for each transition type across mice. Preference Scores are a novel way to normalize transition probabilities that considers the probability of observing a given transition by chance and comparing against what would be predicted if that transition were an absolute rule (see Methods for more details). Conceptually, a Preference Score represents the strength of the preference for a given transition, with a score of 1 being maximally preferred, a score of −1 being maximally dispreferred, and a score of 0 meaning the preference is equal to chance. Crucially, Preference Scores are not biased by individually differences in syllable inventory, and therefore these values provide a method for comparing across mice who do not produce identical proportions of each syllable class.

A comparison of the Preference Scores for each transition across genotypes revealed no significant differences between WT and Foxp2+/− song. Both genotypes preferred to start and end bouts and groups with short syllables and to produce series of short and long syllables, while transitioning between syllable classes was dispreferred (Fig. 4d,e, Supplementary Table S1). Therefore, the rhythmic abnormalities observed in Foxp2+/− song are the byproduct of an abnormal rhythmic syllable inventory, and *not* the result of distorted or incorrectly applied abstract rules.

## Conclusion

In conclusion, we have demonstrated that heterozygous *Foxp2* KO causes severe abnormalities in the development and production of several temporal aspects of adult male mouse courtship song. Interestingly, the effects of heterozygous *Foxp2* KO in adults are much more striking than those previously reported in pups^15^,^16^,^17^,^19^. While the underlying cause of this disassociation remains unclear, it may suggest that either the consequences of heterozygous *Foxp2* KO worsens as mice progress through early postnatal development prior to weaning (<P21), which was not examined in this study, or that mouse courtship song relies on different neural pathways than innate pup calls, as previously proposed^29^,^30^. An additional question raised by this study is whether any aspect of the changes observed in the vocalizations over time are the result of vocal learning, or instead are simply the byproducts of innate developmental processes.

Foxp2+/− mice were found to display specific abnormalities in the production of long syllables and long sequences of syllables, though syllable acoustics were not affected. Foxp2+/− animals also produced song with rhythmic distortions due to abnormalities in their rhythmic syllable inventory rather than deficiencies in abstract rhythmic rule application. Interestingly, humans with *FOXP2* mutations have also been noted to display strong prosodic and speech timing irregularities. Our results suggest that the rhythmic defects present in the vocalizations of humans and mice due to *FOXP2*/*Foxp2* mutation may be due to a common motor deficit that is not specific to language. While further research is required to determine the biological basis of this underlying deficit and resulting vocal phenotype, the present findings suggest that Foxp2+/− mice may have a deficiency in motor planning and the sequencing of rhythmic motor behavior, a hypothesis that is supported by the non-vocal motor learning defects previously observed in *Foxp2* mutant mice^17^,^31^. While *Foxp2* is expressed in the cortex of humans and mice^32^, cortex does not appear to be recruited in the production of mouse song^33^ (but see Arriaga *et al.*, 2012); instead the abnormalities observed in the basal ganglia and cerebellum of *Foxp2* mutant animals^15^,^16^,^17^,^18^ may drive the observed vocalization deficits. Therefore, further study of *Foxp2* mutant mice and their vocalizations may provide valuable insights into the role of these brain regions in generating the coordinated rhythmic orofacial motor behavior that is essential for human speech production.

## Methods

### Animals

Five litters of male Foxp2+/− mice (n = 11) and their male C57Bl/6J wildtype littermates (n = 14) were used as subjects for the study; this line of Foxp2+/− mice are described in Shu *et al.* (2005). Sires were removed from the breeding cage prior to the opening of the ear canals to ensure that none of the subject mice had experience with adult song before the start of the experiment. At weaning (P21), the subject mice were individually housed as to limit their exposure to other mice, and therefore confine their exposure to and production of courtship song, to the socialization sessions. For the data presented in Supplementary Figure S2, 21 male C57Bl/6J mice were ordered from Jackson Laboratories and arrived before P25. Upon arrival, each of the mice were also individually housed. All procedures used in the study were approved by the Institutional Animal Care and Use Committee of Yale University, and were carried out in accordance with relevant guidelines and regulations.

### Vocalization Recording

The Foxp2+/− subject mice and their WT littermates were socialized with a random WT female mouse ~2–3 times a week starting shortly after weaning (between P23 and P29) and concluding well into adulthood (between P70 and P74). During these socialization sessions, a subject mouse was placed in a rectangular recording chamber (approximately 11.5 × 7 inches) with the random female, and the animals were allowed to move and interact freely. The chamber was lined with non-absorbent rubber to dampen the sound of footfalls and allow for removal of odors between sessions. The recording chamber was then placed under an ultrasonic microphone (CM16/CMPA, Avisoft Bioacoustics, Berlin, Germany) in a sound-attenuated booth (Industrial Acoustics, New York, USA). The vocalizations produced during the socialization session were sampled at 250 kHz and digitized using an Avisoft signal conditioner and recorded with the Avisoft RECORDER software. Socialization sessions were at least 3 minutes in duration, and lasted on average 235 seconds +/− 71 seconds (mean +/− 1 standard deviation). Between sessions, the recording cage was cleaned with 70% aqueous ethanol, rinsed with water, and dried.

The C57Bl/6J mice whose data are presented in Supplementary Figure S2 were socialized using the same protocol as above. However, these animals were socialized until P95, and their age during the first socialization session and the frequency of their social exposure was varied: 8 mice were socialized 3 times a week starting at P25, 3 mice were socialized 2 times a week starting at P25, 5 mice were socialized 3 times a week starting at P38, and 5 mice were socialized 3 times a week starting at P60.

Female mice vocalize when paired with male mice^34^,^35^, though at a rate much lower than males^24^,^35^,^36^. Past studies have provided inconclusive evidence for the actual vocalization rate of female mice when interacting with male mice, with some studies reporting that female mice rarely vocalize in such contexts^24^,^36^, while another reported that up to 18% of vocalizations recorded during male-female social pairing are produced by the female^35^. We rarely observed instances where both mice vocalized at the same time during a recording, suggesting that the female mice in our study did indeed vocalize at a much lower rate, consistent with previous reports^24^,^36^, and therefore the vast majority of the vocalizations we recorded were produced by the male mice. Recording multiple sessions of male courtship song over several weeks in response to awake female mice allowed us to accurately characterize naturalistic vocal behavior while also observing its intrinsic variability.

### Acoustical Analysis

We detected and analyzed vocalizations using a custom Matlab (Mathworks, Natick, USA) script. First, we calibrated our detection parameters on a set of six typical recordings (3 WT, 3 Foxp+/−). After bandpass filtering the recordings from 30 to 120 kHz, we pooled all of the instantaneous measures of acoustic power (in full scale relative dBs; dBFS), but were unable to find clear separation between the distributions corresponding to silent and non-silent periods (Supplementary Figure S1a). We then calculated spectral flatness (the geometric mean of the power spectrum divided by its arithmetic mean) in 256 point (1 ms) windows of the recordings with no overlap and found that, while spectral flatness and acoustic power and highly correlated (Supplementary Figure S1c), silent periods could be delineated from periods of vocalization at a cutoff value of 0.6 (Supplementary Figure S1b). We then empirically defined the minimum intersyllable interval (ISI) by examining all silent periods longer than 10 ms in the six recordings (Supplementary Figure S1d). The distribution of these silent periods had a clear trough at 40 ms corresponding to the minimum ISI. In agreement with Sirotin *et al.* (2014), using a value less than 40 ms would therefore result in incorrectly separating single syllables produced on breathing cycle. To summarize, 256 point time windows with spectral flatness values of 0.6 or less separated by less than 40 ms were grouped together into syllables. In addition, any syllable with a dominant frequency less than 39 kHz was rejected, because we found that audible noise in the recordings would occasionally have some power remaining in the passband after filtering, but the dominant frequencies of such sounds were usually below 39 kHz.

Rodent ultrasonic vocalizations are produced via a laryngeal whistle mechanism^37^, resulting in syllables having distinct tonal characteristics and therefore prominent dominant frequencies (Fig. 1a). The shape of a syllable’s dominant frequency trajectory in time (contour shape) has been used to classify syllables and calculate acoustic measurements in many studies^21^,^22^,^23^,^27^,^28^,^29^,^30^,^38^,^39^. Likewise, we identified the contour shape for each syllable by calculating the power spectrum throughout the duration of the syllable in windows of 512 points with 50% overlap and determining the frequency with the highest power in each window, thus providing dominant frequency measurements at approximately 1 ms resolution. We then detected periods of noise within syllables by finding points of unstable dominant frequency, specifically where the dominant frequency changed by more than 10 kHz twice in less than 4 ms. Any such points were rejected from the syllable’s contour shape. This constraint also defined the minimum syllable duration as 4 ms, within the range used in previous studies (e.g. Chabout *et al.*, 2015; Hammerschmidt *et al.*, 2015). Subjectively, this method of determining contour shape was found to work as well as manually labelling the shape, but did so in an unbiased way rather than forcing syllables into predetermined classes. Lastly, we also calculated the number of pitch jumps in each syllable (how many times the dominant frequency contour changed from one point to the next by more than 10 kHz, the cutoff used by Arriaga *et al.*, 2013) and the overall syllable dominant frequency from the power spectrum of the entire syllable.

### Data Analysis

#### Age Grouping

Data from each mouse were separated into three age groups: 1) Juvenile, P20–P34, 2) Intermediate, P35–P49, and 3) Adult, P50–P75. An additional group of Foxp2+/− animals was also considered, which consisted of only data from adult mice who vocalized at a syllable rate of 90 syllables per minute or more (n = 6 mice). All adult WT mice produced at least 90 syllables per minute, so an additional control group for the genotype was not necessary.

#### Syllable Rate

Session syllable rates were calculated by dividing the number of syllables detected in a recording session by the length of that recording. Average syllable rates for age groups were calculated by first dividing the total number of syllables produced by each mouse during all recordings at a given age by the total amount of time the mouse was recorded at that age, and then calculating the average across mice.

#### Duration Measures

To illustrate syllable duration distributions across entire genotypes (the histograms in Fig. 2b,c), we randomly down-sampled the data set from each mouse as to provide an equal number of duration measures per mouse (each mouse contributed the same number of measures as the mouse providing the least data points). Combining these down-sampled measures into one pool therefore provided a genotype-wide distribution that equally weighted each mouse. As the genotype-wide syllable duration distribution for WT mice was clearly bimodal, with one distribution of short duration syllables and another of long duration syllables, we selected the approximate center of the trough between the distributions (75 ms) to operationally define the cut-off between the two syllable classes. Therefore, short syllables have a duration of less than 75 ms and long syllables have a duration of at least 75 ms. The average proportion of long syllables for an age group was calculated by dividing the total number of long syllables produced by each mouse in all recordings at that age by the total number of syllables produced. The mean across all mice was then computed to provide a final age group average.

Average Ashman’s D scores^40^ for each age group were calculated in a similar way. We first fit each mouse’s distribution of syllable duration values (maximum of 300 ms to exclude outliers) with two Gaussians using Matlab’s ‘gmdistribution.fit’ function with a maximum of 500 iterations. To quantify the separation of the two Gaussians, we calculated Ashman’s D scores with the following:


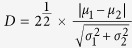


where μ and σ are the center and standard deviation of each Gaussian, respectively. Ashman’s D scores were computed for each animal at each age, and the average across mice provided age and genotype-wide values. From the fit of two Gaussians, the average mu value (center) of each Gaussian as well as the average weight of the longer duration Gaussian was also calculated at each age group across WT mice. For the fitting analyses, one WT animal was excluded because it did not have sufficient data points as a juvenile to constrain the fits. As replicates are required for repeated measures statistics, this animal’s intermediate and adult age group data were also excluded.

#### Sequencing Measures

To examine the distribution of ISIs, we pooled the durations of all ISIs produced in every recording, regardless of age or genotype. The resulting distribution displayed a non-uniform distribution, consistent with previous findings^28^. Using the troughs in this distribution, we operationally defined syllable boundaries as silent periods of at least 40 ms but not more than 150 ms, group boundaries as silent periods of at least 150 ms but not more than 300 ms, and bout boundaries as silent periods of at least 300 ms.

To provide analyses of the number of syllables per group, syllables per bout, and groups per bout (the beeswarm plots in Fig. 3c–e), we randomly down-sampled the data set from each mouse in the same manner as described above for the syllable duration distributions. However, in order to present distributions with a sufficiently large number of data points at each age, mice that produced less than 50 sequences of a given type were excluded from the distribution of corresponding sequencing variable. We did this three times, once for each of the above variables, and combined these down-sampled measures into one pool in order to provide age group-wide distributions for both genotypes that equally weighted each mouse.

The average proportion of long groups and bouts for age groups were calculated by first dividing the number of groups and bouts produced by a mouse at a given age that contained more than the median number of syllables per sequence type (3 syllables per group, and 5 syllables per bout) by the total number of the corresponding sequence type produced by the mouse at that age group. As described above, averaging across mice provided a mean proportion of long groups and bouts for the age group. The same method was used in calculating the proportion of multigroup bouts (at least 2 groups per bout).

#### Transition Probabilities

Conditional transition probabilities were calculated for the following:

1. At the start of a bout, the probability of observing a short syllable or long syllable (b → S or L)
2. At the start of a group, the probability of observing a short syllable or long syllable (g → S or L)
3. At the end of a bout, the probability of observing a short syllable or long syllable (S or L → b)
4. At the end of a group, the probability of observing a short syllable or long syllable (S or L → g)
5. Within a group (transitions to boundaries not considered), the probability of observing a short syllable or long syllable after producing a short syllable (S → S or L)
6. Within a group (transitions to boundaries not considered), the probability of observing a short syllable or long syllable after producing a long syllable (L → S or L)

In each of the 6 situations above, the combined probability of observing either a short syllable or a long syllable adds to 1. As only two transitions are possible in each of the 6 starting points, these conditional probabilities allow for straightforward comparisons between different transition types.

Conditional transition probabilities were normalized by first subtracting the observed conditional probability of a transition by the probability of observing that transition by chance. This difference provided a relative measure of the strength of that transition compared to chance, and determines whether not a transition is preferred (probability greater than chance) or dispreferred (probability less than chance). Absolute “rules” would have conditional probabilities of, or extremely close to, either 1 or 0, consequently the maximum difference from chance a given transition could have would be 1 subtracted by chance probability for a preferred transition, or 0 subtracted by chance for a dispreferred transition. Dividing the observed difference from chance by the maximal difference from chance therefore yields a proportion which represents the strength of the preference for a given transition relative to an absolute rule. Crucially, this value is not biased by individual differences in the proportions of each element within a mouse’s inventory. We refer to this value as a Preference Score; by convention, a preferred transition has a positive Preference Score and a dispreferred transition has a negative Preference Score. A formulaic representation is presented below:


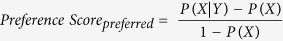



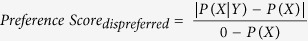


where P(X|Y) is the conditional probability of observing X given Y, and P(X) is the chance probability of observing X in that environment.

Conditional transition probabilities and Preference Scores were calculated for each adult WT and adult Foxp2+/− (SR > 90) mouse. We then averaged across mice to provide mean values for each genotype.

#### Acoustical Measures

To analyze syllable acoustics, we calculated one measure of frequency modulation (the standard deviation of contour dominant frequencies within a syllable) and one of amplitude modulation (the standard deviation of mean relative amplitude within a syllable), as well as two gross spectral measures (syllable dominant frequency and the number of pitch jumps in a syllable). We first calculated within-mouse averages and then computed the mean across mice for syllable-wide values (in Table 1) or genotype-wide values (in Table 2) for each variable.

#### Statistical Analysis

For each comparison across age groups and genotypes, we performed a two-way repeated measures ANOVA with multiple comparisons across age groups but within genotypes, and another with multiple comparisons across genotypes but within age groups. Sidak’s Correction for multiple comparisons was applied for all two-way ANOVAs. For comparisons between the distributions of syllables per group, syllables per bout, and groups per bout across genotypes and age groups, we used one-way Kruskal-Wallis tests to establish significance between age groups and genotypes, as the distributions were highly non-normally distributed. To correct for multiple comparisons in this situation, we used Dunn’s Correction. Adult WT, Foxp2+/−, and Foxp2+/− (SR > 90) groups were compared with single one-way ANOVAs with Tukey’s Correction for multiple comparisons. Lastly, to compare between adult WT syllable classes and between adult WT and Foxp2+/− (SR > 90) syllables, we used Welch’s Unpaired T-Test and corrected for multiple comparisons using the Sidak method. All statistical tests were performed in Graphpad Prism (La Jolla, California, USA).

## Additional Information

**How to cite this article**: Castellucci, G. A. *et al.* Knockout of *Foxp2* disrupts vocal development in mice. *Sci. Rep.* **6**, 23305; doi: 10.1038/srep23305 (2016).

## Supplementary Material

Supplementary Information

## Figures and Tables

**Table 1:** Acoustic Differences between Long and Short Syllables in Adult WT Song.

Acoustic Differences between Long and Short Syllables in Adult WT Song		
Measure	Class	Mean (±1 SEM) Value
Standard Deviation of within-Syllable Dominant Frequency	Short	6,737 (± 207.1) Hz
	Long	11,494 (± 335.5) Hz
Standard Deviation of within-Syllable Relative Amplitude	Short	10.17 (±0.05) dBFS
	Long	10.57 (±0.06) dBFS
Number of Pitch Jumps in a Syllable	Short	0.6654 (±0.03)
	Long	3.116 (±0.18)
Syllable Dominant Frequency	Short	77,554 (±762.2) Hz
	Long	68,302 (±1,349) Hz

All between syllable-class differences are significant, p < 0.0001, using Welch’s Unpaired T-Test with Sidak’s Correction for multiple comparisons; additional statistical details are presented in Supplementary Table S3.

**Table 2:** Lack of Acoustic Differences between Adult WT and Foxp2+/− Song Syllables.

Lack of Acoustic Differences between Adult WT and Foxp2+/− (SR > 90) Song Syllables			
Measure	Class	Genotype	Mean (±1 SEM) Value
Standard Deviation of within-Syllable Dominant Frequency	Short	WT	6,737 (±207.1) Hz
		Foxp2+/−, SR > 90	5,937 (±445.4)Hz
	Long	WT	11,494 (±335.5) Hz
		Foxp2+/−, SR > 90	10,401 (±682.3) Hz
Standard Deviation of within-Syllable Relative Amplitude	Short	WT	10.17 (±0.05) dBFS
		Foxp2+/−, SR > 90	10.13 (±0.07) dBFS
	Long	WT	10.57 (±0.06) dBFS
		Foxp2+/−, SR > 90	10.55 (±0.09) dBFS
Number of Pitch Jumps in a Syllable	Short	WT	0.6654 (±0.03)
		Foxp2+/−, SR > 90	0.6827 (±0.09)
	Long	WT	3.116 (±0.18)
		Foxp2+/−, SR > 90	2.353 (±0.19)
Syllable Dominant Frequency	Short	WT	77,554 (±762.2) Hz
		Foxp2+/−, SR > 90	76,508 (±1,768) Hz
	Long	WT	68,302 (±1,349) Hz
		Foxp2+/−, SR > 90	68,385 (±2,301) Hz

No between-genotype differences are significant using Welch’s Unpaired T-Test with Sidak’s Correction for multiple comparisons; additional statistical details are presented in Supplementary Table S3.
