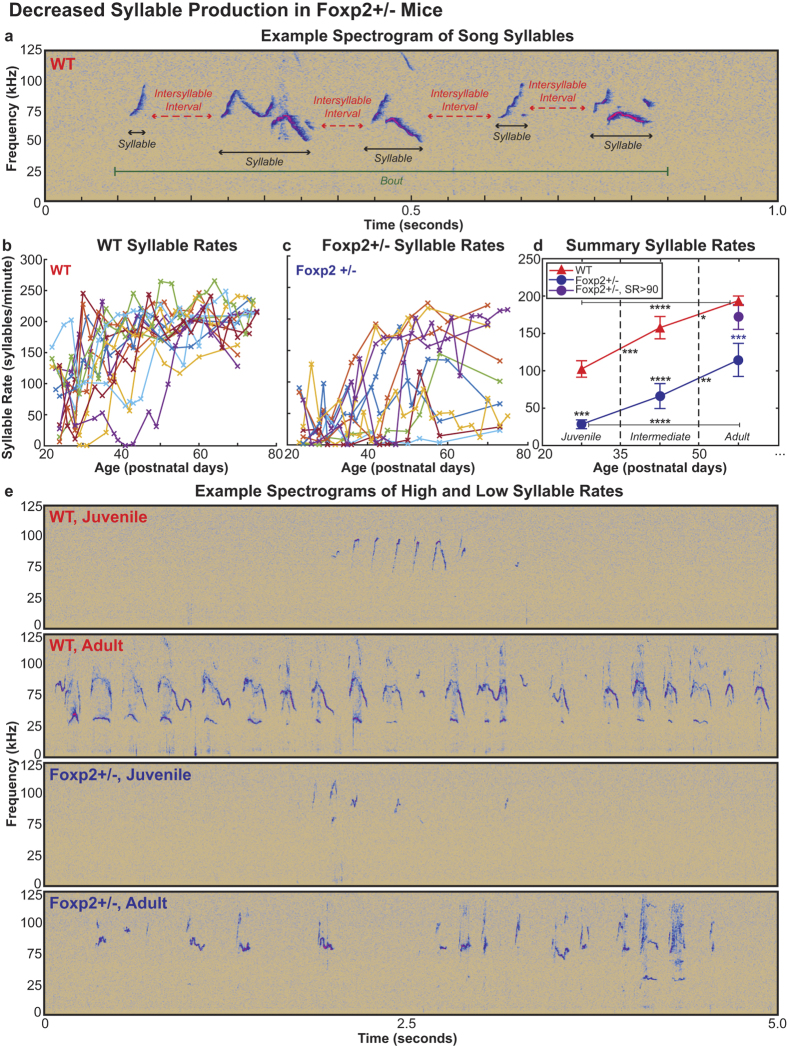# Corrigendum: Knockout of *Foxp2* disrupts vocal development in mice

**DOI:** 10.1038/srep39722

**Published:** 2017-01-12

**Authors:** Gregg A. Castellucci, Matthew J. McGinley, David A. McCormick

Scientific Reports 6: Article number: 2330510.1038/srep23305; published online: 03 16 2016; updated: 01 12 2017

In this Article there is a plotting error in Figure 1b and c, due to the data sorting technique. The correct figure appears below as Figure 1. This error in no way affects other analyses, results, or the conclusions of this paper.

## Figures and Tables

**Figure 1:**