# Supplementary material for: Knockout of Foxp2 disrupts vocal development in mice
Source: Sci Rep. 2016 Mar 16;6:23305. doi: 10.1038/srep23305 (PMC4793191; doi:10.1038/srep23305)
Supplement: Supplementary Information [file srep23305-s1.pdf]

Knockout of *Foxp2* disrupts vocal development in mice.

Authors: Gregg A. Castellucci[1,2,3], Matthew J. McGinley[1], David A. McCormick[1*]

[1]Yale School of Medicine, Department of Neuroscience, New Haven, 06519, USA

[2]Yale University, Department of Linguistics, New Haven, 06520, USA

[3]Haskins Laboratories, New Haven, 06511, USA

*Correspondence and requests for materials should be addressed to D.M. (david.mccormick@yale.edu).

Supplementary Information - Supplemental Figures and Figure Legends

Supplementary Figure S1 Syllable detection parameters. From 6 typical recordings of mouse song (3 WT, 3 Foxp2+/-): **(a)** histogram of all instantaneous full-scale decibel (dBFS) measurements of acoustic power, **(b)** histogram of spectral flatness values measured in 1 ms bins from the same six recordings with the maximum spectral flatness value of 0.6 used in this study indicated, **(c)** spectral flatness plotted against the average dBFS values in 1 ms bins, and **(d)** histogram of all silent periods at least 10 ms in duration; the minimum ISI value of 40 ms used in this study is indicated.

Supplementary Figure S2 Vocalization Production is Dependent on Socialization History. Syllable rate (syllables per minute) in each recording session for WT mice **(a)** socialized 3 times a week from P25 to P95 (n = 8 mice), **(b)** socialized 2 times a week from P25 to P95 (n = 3 mice), **(c)** socialized 3 times a week from P38 to P95 (n = 5 mice), and **(d)** socialized 3 times a week from P60 to P95. Each line represents a different mouse.

Supplementary Figure S3 Gaussian Fits of WT Syllable Duration Distributions. **(a)** Average Gaussian centers from the fit of two Gaussians to each WT mouse's syllable duration distribution at each age. **(b)** Average fit weights across WT mice for the Gaussians corresponding to longer duration syllables from the fit of two Gaussians described in (a). In (a) and (b), data are presented as means +/- 1 SEM. All significant differences between age groups are indicated (*, $p < 0.05$; ** $p < 0.005$; *** $p < 0.0005$; ****, $p < 0.0001$; two-way repeated measures ANOVA with Sidak's Correction for multiple comparisons. Additional statistical details are presented in Supplementary Table S1.

Supplementary Table S1 ANOVA Statistics.

Supplementary Table S2 Kruskal-Wallis Statistics.

Supplementary Table S3 Welch's T-Test Statistics.

Syllable Detection Parameters

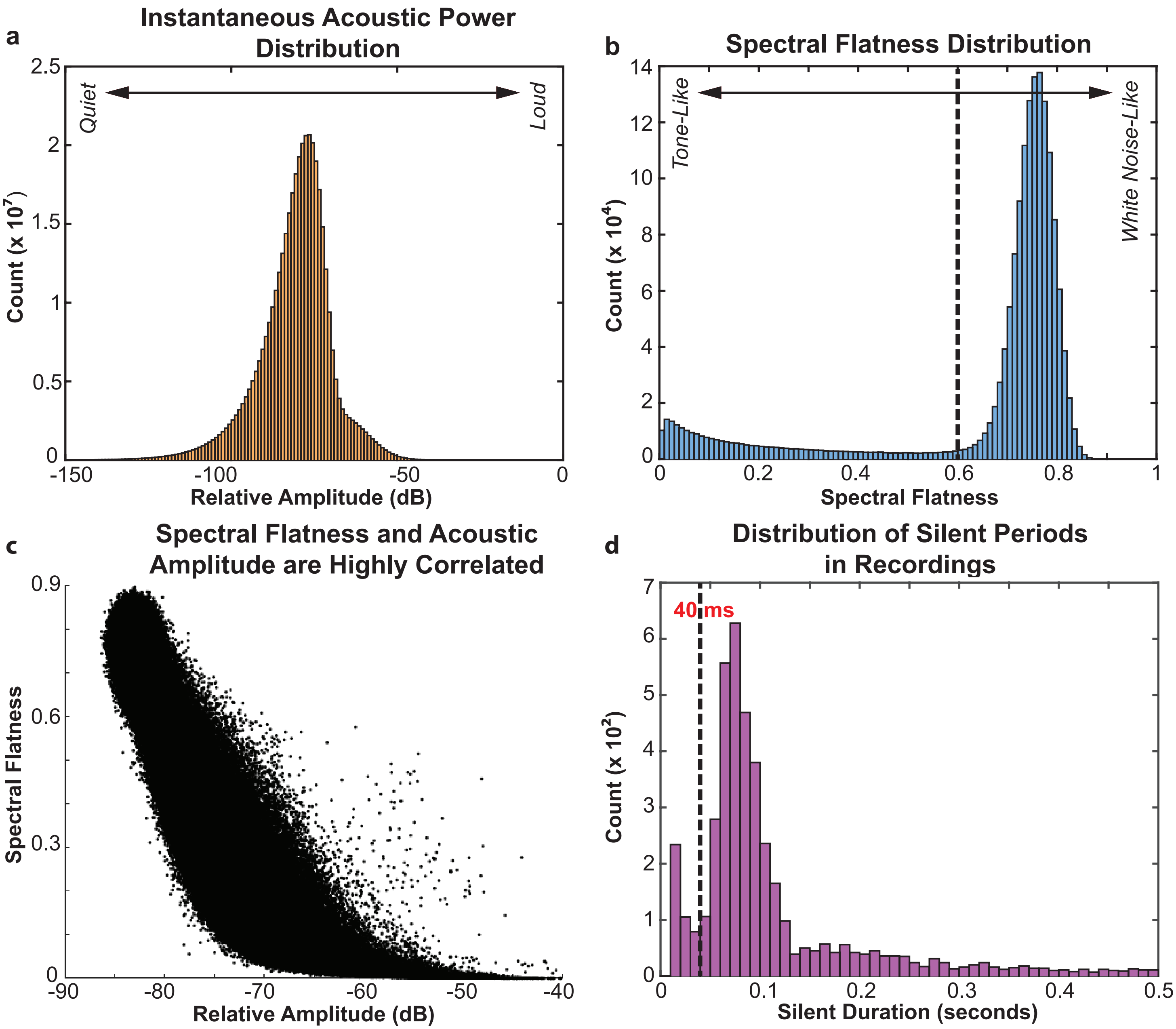

Vocalization Production is Dependent on Socialization History

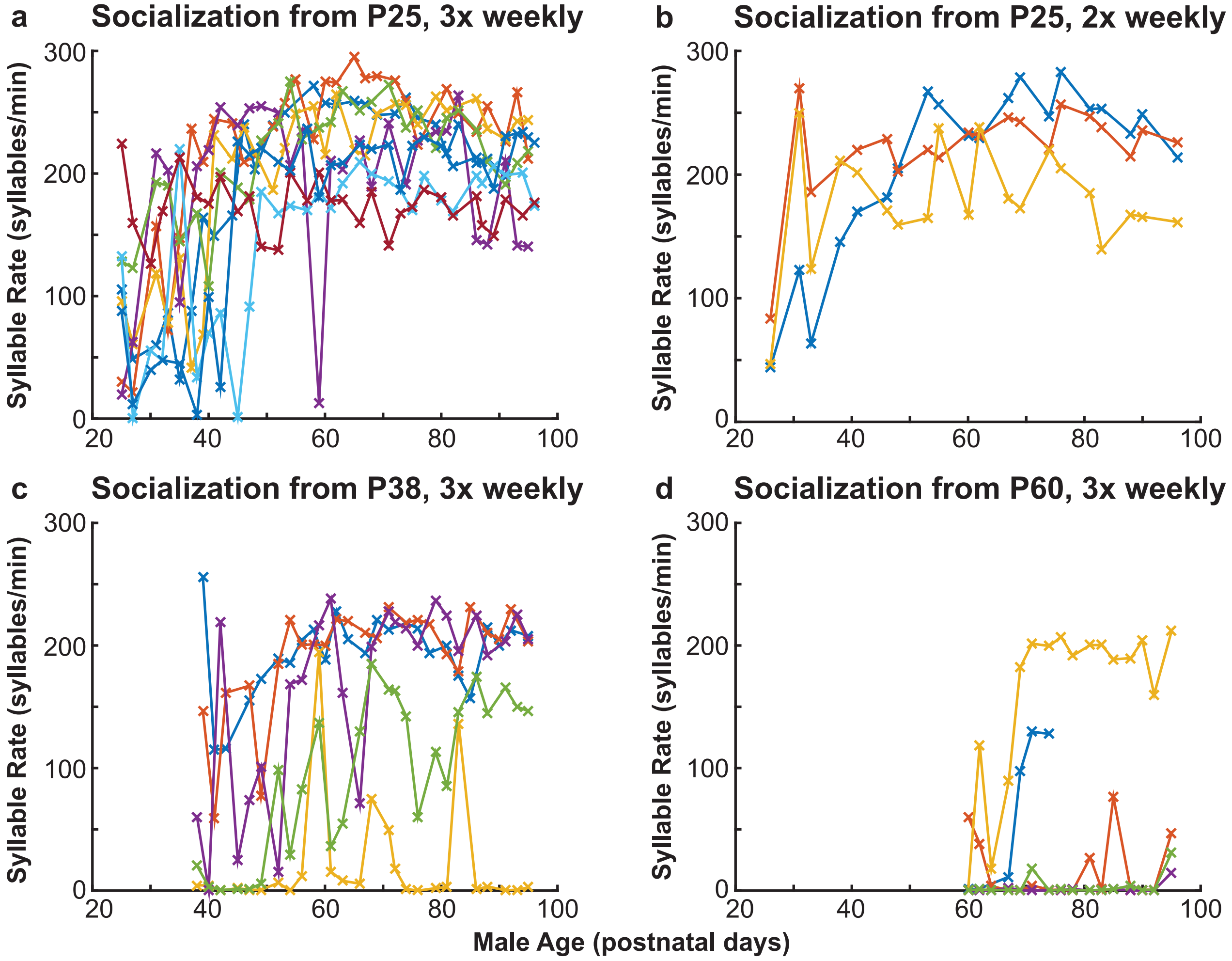

Gaussian Fits of WT Syllable Duration Distributions

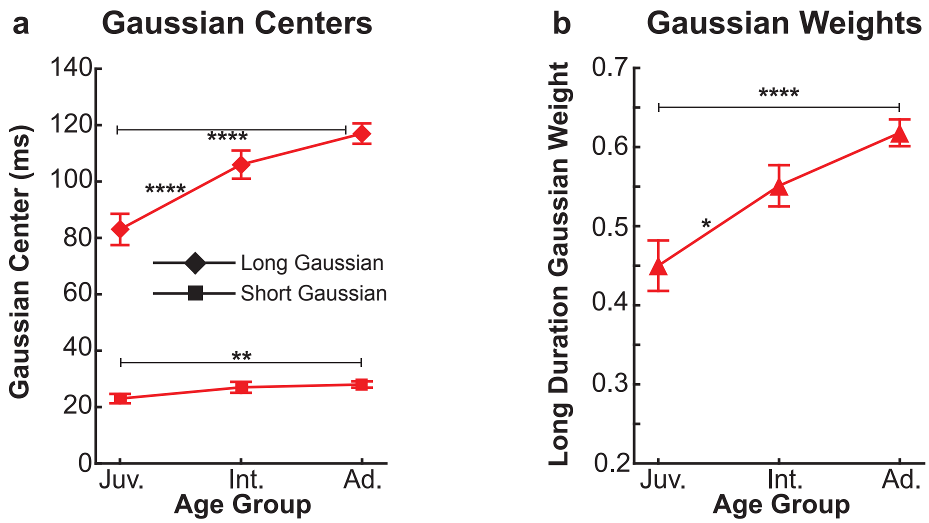

ANOVA Statistics*

Figure	Test Used	Source of Variation	P-Value	F-Value	Sample Size		
					WT	Foxp2+/-	Foxp2+/ (SR>90)
1d	2-way repeated measures ANOVA; Sidak's Correction	Interaction	0.6807	F (2, 46) = 0.3879	14 mice	11 mice	N/A
		Genotype	<0.0001****	F (1, 23) = 28.94			
		Age	<0.0001****	F (2, 46) = 37.06			
		Subjects (matching)	0.003***	F (23, 46) = 3.248			
	1-way ANOVA; Tukey's Correction (between Adult groups)	Between Groups	0.0018***	F (2, 28) = 7.948			6 mice
2e	2-way repeated measures ANOVA; Sidak's Correction	Interaction	0.071	F (2, 44) = 2.810	14 mice	11 mice	N/A
		Genotype	0.081	F (1, 22) = 3.345			
		Age	<0.0001****	F (2, 44) = 22.78			
		Subjects (matching)	<0.0001****	F (22, 44) = 4.293			
	1-way ANOVA; Tukey's Correction (between Adult groups)	Between Groups	0.6523	F (2, 27) = 0.4341			6 mice
2f	2-way repeated measures ANOVA; Sidak's Correction	Interaction	0.0004***	F (2, 44) = 9.290	13 mice	11 mice	N/A
		Genotype	<0.0001****	F (1, 22) = 27.29			
		Age	0.063	F (2, 44) = 2.946			
		Subjects (matching)	<0.0001****	F (22, 44) = 6.460			
	1-way ANOVA; Tukey's Correction (between Adult groups)	Between Groups	<0.0001****	F (2, 27) = 36.25			6 mice
S3b, Short Gaussian	2-way repeated measures ANOVA; Sidak's Correction	Interaction	0.071	F (2, 44) = 2.810	13 mice	11 mice	N/A
		Genotype	0.081	F (1, 22) = 3.345			
		Age	<0.0001****	F (2, 44) = 22.78			
		Subjects (matching)	<0.0001****	F (22, 44) = 4.293			
	1-way ANOVA; Tukey's Correction (between Adult groups)	Between Groups	0.6523	F (2, 27) = 0.4341			6 mice
S3b, Long Gaussian	2-way repeated measures ANOVA; Sidak's Correction	Interaction	0.1414	F (2, 44) = 2.045	13 mice	11 mice	N/A
		Genotype	<0.0001****	F (1, 22) = 44.81			
		Age	<0.0001****	F (2, 44) = 30.11			
		Subjects (matching)	0.0006**	F (22, 44) = 3.156			
	1-way ANOVA; Tukey's Correction (between Adult groups)	Between Groups	<0.0001****	F (2, 27) = 34.52			6 mice
S3c	2-way repeated measures ANOVA; Sidak's Correction	Interaction	0.1269	F (2, 44) = 2.164	13 mice	11 mice	N/A
		Genotype	0.0002***	F (1, 22) = 19.88			
		Age	0.0001***	F (2, 44) = 10.98			
		Subjects (matching)	0.0002***	F (22, 44) = 3.503			
	1-way ANOVA; Tukey's Correction (between Adult groups)	Between Groups	0.0002***	F (2, 27) = 11.54			6 mice
3f	2-way repeated measures ANOVA; Sidak's Correction	Interaction	0.0149*	F (2, 46) = 4.615	14 mice	11 mice	N/A
		Genotype	<0.0001****	F (1, 23) = 34.29			
		Age	0.0071**	F (2, 46) = 5.512			
		Subjects (matching)	0.0031**	F (23, 46) = 2.581			
	1-way ANOVA; Tukey's Correction (between Adult groups)	Between Groups	<0.0001****	F (2, 28) = 26.48			6 mice
3g	2-way repeated measures ANOVA; Sidak's Correction	Interaction	0.1725	F (2, 46) = 1.826	14 mice	11 mice	N/A
		Genotype	<0.0001****	F (1, 23) = 64.50			
		Age	0.0003***	F (2, 46) = 9.939			
		Subjects (matching)	0.0329*	F (23, 46) = 1.890			
	1-way ANOVA; Tukey's Correction (between Adult groups)	Between Groups	<0.0001****	F (2, 28) = 23.82			6 mice
3h	2-way repeated measures ANOVA; Sidak's Correction	Interaction	0.9507	F (2, 46) = 0.05062	14 mice	11 mice	N/A
		Genotype	0.0003***	F (1, 23) = 17.80			
		Age	0.0005***	F (2, 46) = 8.905			
		Subjects (matching)	0.0017**	F (23, 46) = 2.760			
	1-way ANOVA; Tukey's Correction (between Adult groups)	Between Groups	0.0041**	F (2, 28) = 6.732			6 mice
4b,4c	1-way ANOVA; Tukey's Correction (between Adult groups)	Between Groups	<0.0001****	F (23, 216) = 116.2	14 mice	N/A	6 mice
4d,4e	1-way ANOVA; Tukey's Correction (between Adult groups)	Between Groups	<0.0001****	F (23, 216) = 62.09	14 mice	N/A	6 mice

*P-values for individual multiple comparisons are reported in figures.

Kruskal-Wallis Statistics*												
**Figure**	**Test Used**	**Source of Variation**	**P-Value**	**Kruskal-Wallis Statistic**	**Sample Size**							
					WT			Foxp2+/-				
					Juvenile	*Intermediate*	*Adult*	*Juvenile*	*Intermediate*	*Adult*	*Adult, SR>90*
Figure 3c	1-way Kruskal-Wallis; Dunn's Correction	Between Groups	0.8244	2.874	2301 groups	2240 groups	6160 groups	594 groups	600 groups	608 groups	4542 groups
Figure 3d	1-way Kruskal-Wallis; Dunn's Correction	Between Groups	<0.0001****	-301.1	1287 bouts	1498 bouts	3836 bouts	416 bouts	1179 bouts	1155 bouts	2892 bouts
Figure 3e	1-way Kruskal-Wallis; Dunn's Correction	Between Groups	<0.0001****	-1110	1287 bouts	1498 bouts	3836 bouts	416 bouts	1179 bouts	1155 bouts	2892 bouts

*P-values for individual multiple comparisons are reported in figures.

Welch's T-Tests							
**Figure**	**Comparison**	**Two-Tailed P-Value**	**Welch's Statistics**	**Adjusted Significance Threshold (Sidak Corrected)**		**Sample Size**	
						WT	Foxp2+/-
Table 1	St. Dev. of within-Syllable Dominant Frequency	<0.0001****	t=12.07 df=21.65	0.01	5 Comparisons	14 mice	N/A
	St. Dev. of within-Syllable Relative Amplitude	<0.0001****	t=5.197 df=24.55	0.01	5 Comparisons	14 mice	N/A
	Number of Pitch Jumps	<0.0001****	t=13.80 df=13.70	0.01	5 Comparisons	14 mice	N/A
	Dominant Frequency	<0.0001****	t=5.971 df=20.53	0.01	5 Comparisons	14 mice	N/A
Table 2	St. Dev. of within-Syllable Dominant Frequency, Short Syllables	0.1906	t=1.438 df=7.541	0.0064	8 Comparisons	14 mice	6 mice
	St. Dev. of within-Syllable Dominant Frequency, Long Syllables	0.1459	t=1.628 df=7.265	0.0064	8 Comparisons	14 mice	6 mice
	St. Dev. of within-syllable Relative Amplitude, Short Syllables	0.6015	t=0.5392 df=10.02	0.0064	8 Comparisons	14 mice	6 mice
	St. Dev. of within-syllable Relative Amplitude, Long Syllables	0.8413	t=0.2057 df=9.770	0.0064	8 Comparisons	14 mice	6 mice
	Number of Pitch Jumps, Short Syllables	0.8659	t=0.1763 df=5.956	0.0064	8 Comparisons	14 mice	6 mice
	Number of Pitch Jumps, Long Syllables	0.0091	t=2.975 df=15.61	0.0064	8 Comparisons	14 mice	6 mice
	Dominant Frequency, Short Syllables	0.9756	t=0.03133 df=10.27	0.0064	8 Comparisons	14 mice	6 mice
	Dominant Frequency, Long Syllables	0.6011	t=0.5434 df=8.305	0.0064	8 Comparisons	14 mice	6 mice
